# Evaluation of apathy in non-clinical populations: validation, psychometric properties, and normative data of the Italian version of Apathy-Motivation Index (AMI)

**DOI:** 10.1007/s10072-023-06774-0

**Published:** 2023-04-03

**Authors:** Manuela Altieri, Gianpaolo Maggi, Valentina Rippa, Gabriella Santangelo

**Affiliations:** Department of Psychology, University of Campania “Luigi Vanvitelli”, Caserta, Italy

**Keywords:** Apathy, Motivation, Validation study, Healthy aging, Normative data

## Abstract

**Introduction:**

Evaluation of apathy in non-clinical populations is relevant to identify individuals at risk for developing cognitive decline in later stages of life, and it should be performed with questionnaires specifically designed for healthy individuals, such as the Apathy-Motivation Index (AMI); therefore, the aim of the present study was to validate the AMI in a healthy Italian population, and to provide normative data of the scale.

**Materials and methods:**

Data collection was performed using a survey completed by 500 healthy participants; DAS, MMQ-A, BIS-15, PHQ-9, and GAD-7 were used to investigate convergent and divergent validity. Internal consistency and factorial structure were also evaluated. A regression-based procedure and receiver operating characteristics (ROC) analyses were used to evaluate the influence of socio-demographic variables on AMI scores and to provide adjusting factors and three cut-offs for the detection of mild, moderate, and severe apathy.

**Results:**

The Italian version of the AMI included 17 items (one item was removed because it was not internally consistent) and demonstrated good psychometric properties. The three-factor structure of AMI was confirmed. Multiple regression analysis revealed no effect of sociodemographic variables on the total AMI score. ROC analyses revealed three cut-offs of 1.5, 1.66, and 2.06 through the Youden’s *J* statistic to detect mild, moderate, and severe apathy, respectively.

**Conclusion:**

The Italian version of the AMI reported similar psychometric properties, factorial structure, and cut-offs to the original scale. This may help researchers and clinicians to identify people at risk and address them in specific interventions to lower their apathy levels.

**Supplementary Information:**

The online version contains supplementary material available at 10.1007/s10072-023-06774-0.

## Introduction

Apathy is defined as a reduction in motivation and frequency of goal-directed cognitive, emotional, and/or social activities with respect to a previous level of functioning [1]. As a neuropsychiatric syndrome, apathy occurs in several neurological diseases; positive associations between levels of apathy and cognitive dysfunctions have been observed in mild cognitive impairment [2, 3], dementia [4], Parkinson’s disease [5–7], multiple sclerosis [8, 9], amyotrophic lateral sclerosis [10], Huntington’s disease [11], and stroke [12]. Moreover, apathy has been found to be a risk factor for cognitive decline over time and conversion to frank dementia [13–17], and higher levels of apathy seem to be associated with lower levels of cognitive reserve [18] — the ability of the brain to cope with physiological or pathological brain damage and to protect against dementia in older age [19]. Taking into account these assumptions, it seems relevant to evaluate apathy in non-clinical populations to identify early apathetic individuals at high risk of developing cognitive decline.

Apathetic behavior has been evaluated using different tools such as the Apathy Evaluation Scale [20], Starkstein Apathy Scale [21], Apathy Inventory [22], Lille Apathy Rating Scale [23, 24], and Dimensional Apathy Scale [25]. Most of these questionnaires were adapted and validated for the Italian population [26–29] with mixed levels of study quality [30]. These tools were originally conceived for use in clinical populations, especially in patients with neurological conditions. The few questionnaires developed for the general population do not specifically assess apathetic behaviors, but they focus only on levels and types of motivation; moreover, cut-off values for lack of motivation are not provided [31].

Recently, a novel self-report questionnaire, the Apathy-Motivation Index (AMI) [32], was developed to fill this gap in the literature and to be employed in healthy people. AMI was created by a team of clinical neurologists and researchers with expertise in apathy, taking into account the multidimensional approach provided by a structured interview for neurological patients, the Lille Apathy Rating Scale [23, 24], and creating a set of items reflecting three dimensions of apathy/lack of motivation: behavioral activation, social motivation, and emotional sensitivity. The exploratory factor analysis identified a clear three-factor structure, starting from a preliminary 51-item scale. The authors retained the six highest loading items for each factor, providing a final version of 18 items. The construct validity and internal reliability of AMI were adequate and acceptable; moreover, cut-off values were provided to identify people with moderate and severe apathy.

Considering that the presence of higher levels of apathy in otherwise healthy people may interfere not only with their quality of life and educational and job opportunities but also directly or indirectly increase the risk of dementia in later stages of life [18, 33, 34], a timely identification of people at risk may help to address these people to non-pharmacological programs designed to increase levels of motivation and counteract the effect of mild, moderate, or severe apathy, as detected by AMI scores. Therefore, the aim of the present study was to provide psychometric and diagnostic properties, as well as normative data, of the first Italian version of the AMI in an Italian non-clinical population.

## Materials and methods

### Participants

Participants were recruited through an online survey created on Google Forms. Data were acquired from April 20 to June 24, 2021. The survey was disseminated using a snowball sampling strategy to university students and psychologist trainees, who were asked to distribute the online survey to their families, friends, and acquaintances throughout the Italian territory. The link was also shared on social media platforms (i.e., Facebook, Instagram, WhatsApp). Participants who reported being affected by neurological and/or psychiatric diseases or if they used psychotropic drugs at the time of the survey were excluded from the analyses. The study was approved by the Local Ethics Committee and was carried out in accordance with the Declaration of Helsinki.

### Structure of AMI

The original version of the AMI includes 18 items on a 5-point Likert scale ranging from 0 (completely true) to 4 (completely untrue). Every item is negatively scored; thus, higher scores are indicative of more apathetic behavior. The scale includes three domains of apathy-motivation: behavioral activation (items 5, 9, 10, 11, 12, 15), social motivation (items 2, 3, 4, 8, 14, 17), and emotional sensitivity (items 1, 6, 7, 13, 16, 18). The score for each domain was obtained using the following formula: $\frac{\mathrm{sum}\;\mathrm{of}\;\mathrm{items}\;\mathrm{of}\;\mathrm{the}\;\mathrm{specific}\;\mathrm{domain}}{\mathrm{number}\;\mathrm{of}\;\mathrm{items}\;\mathrm{in}\;\mathrm{the}\;\mathrm{specific}\;\mathrm{domain}}$. The total AMI score was computed by averaging the mean scores of the three domains.

### Italian adaptation of AMI

The English version of AMI [32] was independently translated into Italian by two researchers. The two versions were merged into a draft, and possible discrepancies were discussed among the authors to reach an agreement. According to the guidelines of Beaton and colleagues [35], the Italian draft was back translated into English by a native English speaker with expertise in linguistics and psychology. The back-translated and original English versions of the AMI were compared to evaluate the linguistic and psychological equivalence of the two versions. The two versions were defined as being equivalent. Finally, the linguistic comprehensibility of each item of the Italian version of AMI was tested by administering the scale to a group of 25 participants (aged 18–65 years old); no item was judged incomprehensible by participants, and the translated version of AMI was considered final.

### Structure of the survey and psychological-behavioral assessment

The online survey included:

1. Informed consent form: before being able to complete the online survey, every participant needed to provide their consent to participate in the study;
2. A sociodemographic questionnaire aimed at collecting data about sex, age, and years of education for each participant. The possible occurrence of current and/or past neurological and psychiatric diseases and the use of psychotropic drugs were also investigated;
3. The Italian version of AMI;
4. The Dimensional Apathy Scale (DAS) [29] to evaluate the levels of apathy and convergent validity;
5. The ability subscale of the Multifactorial Memory Questionnaire (MMQ-A) [36] to evaluate subjective memory functioning, particularly the frequency of memory problems;
6. The short version of the Barratt Impulsiveness Scale (BIS-15) [37] to assess levels of impulsivity;
7. The 9-item Patient Health Questionnaire (PHQ-9) [38] to evaluate depressive symptomatology;
8. The 7-item Generalized Anxiety Disorder scale (GAD-7) [39] to assess severity of anxiety.

### Statistical analysis

Statistical analyses were performed using IBM SPSS Statistics, version 26. Acceptability of the AMI was defined by low percentages of missing values and floor and ceiling effects, according to previous studies on the standardization of behavioral scales [37, 40].

We checked univariate normality through skewness and kurtosis values. Values not exceeding |2| typically indicate no significant distortions from the Gaussian distribution [41].

The internal consistency was tested by Cronbach’s alpha coefficient. We obtained additional evidence on the reliability and scaling assumptions for each item by computing Pearson’s item-total correlations and corrected item-total correlations to adjust for inflation errors [32, 42]. We interpreted the effect size according to Cohen’s conventions (weak, *r* < 0.30; moderate,* r* = 0.30–0.50; strong, *r* > 0.50) [43].

Confirmatory factor analysis (CFA) was conducted using AMOS. We assessed the model fit using root-mean-square error of approximation (RMSEA), standardized root-mean-square residual (SRMR), and comparative fit index (CFI). We adopted a cut-off of 0.80 for CFI and of < 0.08 for RMSEA and SRMR in line with previous investigations [32].

Convergent validity was assessed by Pearson’s correlation between AMI and DAS total scores, while divergent validity was evaluated by the correlation between the AMI total score and the scores of the PHQ-9, GAD-7, BIS-15, and MMQ. We also evaluated the potential influence of demographic factors (i.e., age, educational level, and sex) on the total AMI score through a multiple linear regression analysis.

Diagnostic accuracy was evaluated via receiver operating characteristics (ROC) analysis. A score above the 95th percentile of the DAS total score was employed as the state variable. Intrinsic properties — sensitivity (Se) and specificity (Sp) — were determined, and the optimal cut-off was identified through the Youden’s *J* statistic. Two additional cut-offs were identified for moderate and severe apathy on the AMI to be respectively > 1 SD and > 2 SD above the mean following the original scale [32].

## Results

The online questionnaire was completed by 648 participants. Nevertheless, 148 participants who reported the presence of neurological/psychiatric conditions, cognitive decline, or ongoing treatment with psychotropic drugs were excluded. Hence, the final sample consisted of 500 participants (146 men and 354 women; see also Tables 1 and 2) with a mean age of 37.22 (SD = 13.67) and an average educational level of 15.32 years (SD = 2.52).

**Table 1:** Socio-demographic characteristics and neuropsychological/behavioral assessment of the sample (*N* = 500)

Sex, *N* (M/F)		146/354
Age (years)		37.2 (13.7)
Education (years)		15.3 (2.5)
Geographical localization	Northern Italy	19.4%
	Central Italy	20.4%
	Southern Italy	58.6%
AMI		1.2 (0.4)
MMQ-A		55.5 (12.2)
BIS-15		27.9 (5.7)
PHQ-9		7.2 (3.9)
GAD-7		7.8 (4.1)
DAS		21.8 (7.7)

Unless specified, all values are reported as mean (standard deviation)

*AMI* Apathy-Motivation Index, *MMQ-A* Multifactorial Memory Questionnaire – ability subscale, *BIS-15* 15-item Barratt Impulsiveness Scale, *PHQ-9* 9-item Patient Health Questionnaire, *GAD-7* 7-item General Anxiety Disorder scale, *DAS* Dimensional Apathy Scale

**Table 2:** Normative sample stratified by age, sex, and education (*N* = 500)

	Age (years)						
	18–30	31–40	41–50	51–60	61–70	71 +	Total
Low education							
Men	32	14	8	10	4	3	71
Women	51	32	23	19	11	0	136
High education							
Men	31	14	12	9	7	2	75
Women	103	54	32	20	9	0	218
Total							
Men	63	28	20	19	11	5	146
Women	154	86	55	39	20	0	354

Low education: 0–13 years; high education: > 13 years

No significant differences emerged between males and females on age (*F*_(1, 498)_ = 2.981, *p* = 0.085) and educational level (*F*_(1, 498)_ = 2.120, *p* = 0.146). Each variable under examination did not exceed the normality range |2| for skewness and kurtosis values. No missing data or floor or ceiling effects were detected.

Multiple regression analysis aimed at evaluating the possible effect of sociodemographic variables on AMI total score revealed that the effect of sex, age, and educational level on AMI total score was not significant.

### Reliability

Most of the items of the AMI were moderately (items 2, 3, 4, 8, 9, 10, 11, 12, 13, 14, 15, 16, 17; *r* range = 0.393–0.496, *p*_s_ < 0.001) correlated with the total score (see Table 3). Except for item 8, these items showed an acceptable level of discrimination (corrected item-total correlations, range = 0.287–0.377). Some items (items 1, 5, 7, 18) showed weak-to-moderate correlations (*r* range = 0.232–0.375) with the total score and a low level of discrimination (corrected item-total correlations, range = 0.118–0.261). Otherwise, item 6 was weakly correlated with the total score (*r* = 0.089, *p* = 0.047) and demonstrated a very poor level of discrimination (corrected item-total correlation =  − 0.070). Taken together, these results indicated that item 6 was not internally consistent and deserved to be excluded; thus, we will now refer to a 17-items AMI scale without considering item 6 (for the scale translated into the Italian language see Supplementary Material 1; for the scoring sheet see Supplementary Material 2).

**Table 3:** Apathy-Motivation Index (AMI) item characteristics

	Mean ± SD	Item-total correlation	Corrected item-total correlation	Cronbach’s alpha if item removed
1. I feel sad or upset when I hear bad news	0.96 ± 0.80	0.232	0.118	0.659
2. I start conversations with random people	1.86 ± 1.15	0.435	0.287	0.641
3. I enjoy doing things with people I have just met	2.09 ± 1.03	0.443	0.312	0.638
4. I suggest activities for me and my friends to do	1.11 ± 0.98	0.496	0.377	0.630
5. I make decisions firmly and without hesitation	1.63 ± 1.04	0.299	0.153	0.658
6. After making a decision, I will wonder if I have made the wrong choice	1.62 ± 1.10	0.089	− 0.070	0.686
7. Based on the last two weeks, I would say I care deeply about how my loved ones think of me	1.14 ± 1.01	0.300	0.158	0.657
8. I go out with friends on a weekly basis	2.28 ± 1.38	0.393	0.206	0.656
9. When I decide to do something, I am able to make an effort easily	1.03 ± 0.92	0.448	0.332	0.636
10. I do not like to laze around	1.16 ± 1.13	0.462	0.319	0.636
11. I get things done when they need to be done, without requiring reminders from others	0.96 ± 0.93	0.429	0.311	0.639
12. When I decide to do something, I am motivated to see it through to the end	0.83 ± 0.83	0.409	0.302	0.641
13. I feel awful if I say something insensitive	1.05 ± 0.97	0.416	0.290	0.641
14. I start conversations without being prompted	1.63 ± 1.09	0.448	0.309	0.638
15. When I have something I need to do, I do it straightaway so it is out of the way	1.44 ± 1.00	0.429	0.300	0.639
16. I feel bad when I hear an acquaintance has an accident or illness	0.56 ± 0.74	0.436	0.344	0.638
17. I enjoy choosing what to do from a range of activities	0.91 ± 0.85	0.448	0.342	0.636
18. If I realize I have been unpleasant to someone, I will feel terribly guilty afterwards	0.85 ± 0.86	0.375	0.261	0.645

However, the whole scale demonstrated fair internal consistency, as shown by a Cronbach’s alpha of 0.658, which increased (0.686) with the exclusion of item 6.

### Confirmatory factor analysis

We confirmed the three-factor structure of the AMI with model fit indices almost identical to the original scale (Ang et al., 2017) (RMSEA = 0.073 with 90% CI = 0.065–0.080; SRMR = 0.074; CFI = 0.819).

The first factor included items evaluating the capabilities to feel positive and negative affections such as “I feel sad or upset when I hear bad news” and “Based on the last two weeks, I would say I care deeply about how my loved ones think of me” and thus, loaded under the emotional sensitivity factor. The second factor consisted of items reflecting behavioral activation like “When I decide to do something, I am able to make an effort easily” and “I get things done when they need to be done, without requiring reminders from others.” The third factor was composed of items indicating the level of engagement in social interactions and loading on a *social motivation* dimension such as “I suggest activities for me and my friends to do” and “I start conversations without being prompted.”

### Convergent and divergent validity

Convergent validity was demonstrated by a significant strong correlation between AMI and DAS total scores (*r* = 0.594, *p* < 0.001). Conversely, divergent validity was explored by correlating the AMI scores with the PHQ-9, GAD-7, and BIS-15. We found positive and significant correlations with PHQ-9 (*r* = 0.225, *p* < 0.001) and BIS-15 (*r* = 0.199, *p* < 0.001), and negative and significant association with the ability subscale of MMQ (*r* =  − 0.184, *p* < 0.001); on the other hand, AMI was not associated with GAD-7 (*r* = 0.051, *p* = 0.258).

### Diagnostic accuracy and normative data

We carried out ROC analysis using a score above the 95th percentile on the DAS as gold standard. According to this operationalization, 11 participants (2.2%) were classified as apathetic.

**Fig. 1:**
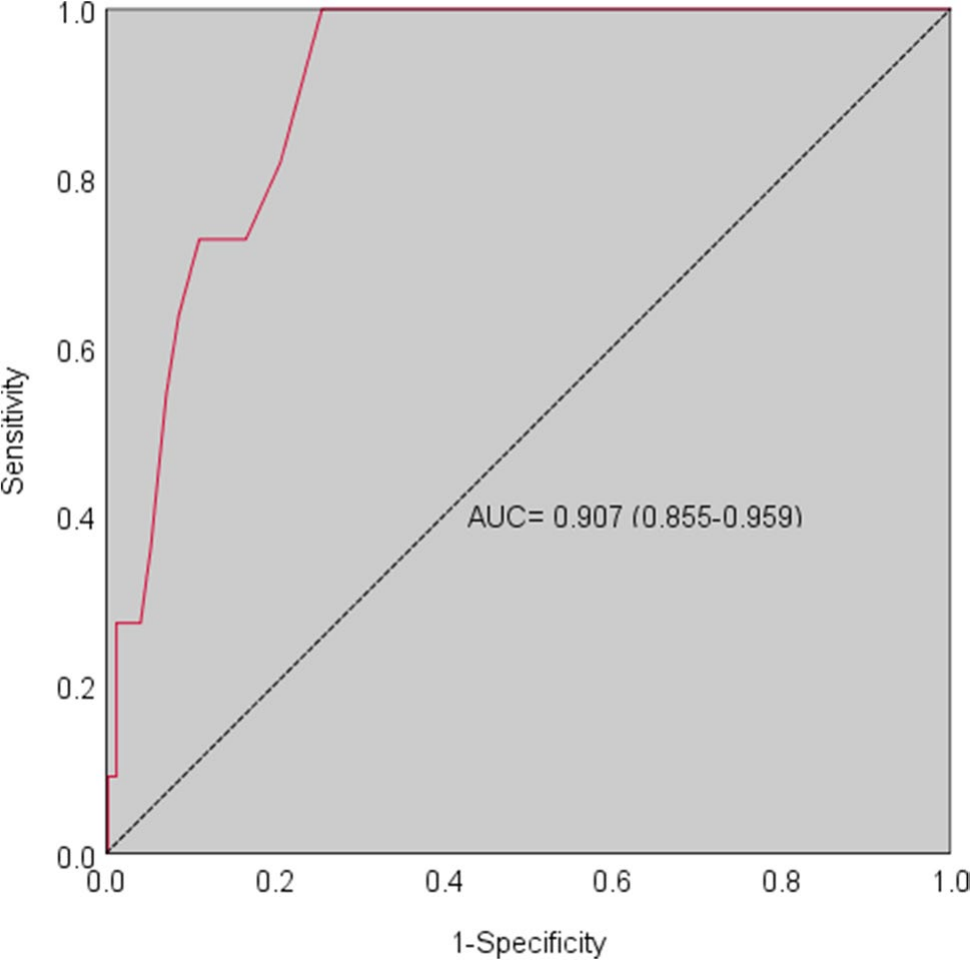
ROC curve for the Italian version of AMI

AMI demonstrated high accuracy in detecting apathetic and non-apathetic individuals (*AUC* = 0.907; *SE* = 0.026; CI 95% [0.855, 0.959]; Fig. 1) with good intrinsic properties (*Se* = 1.000; *Sp* = 0.744). The optimal cut-off score for mild apathy was 1.5 (*J* = 0.744). Based on the original study on AMI [32], we proposed two additional cut-offs to identify moderate (> 1 SD = 1.66) and severe (> 2 SD = 2.06) apathy.

## Discussion

The present study aimed at providing an Italian version of the AMI, a novel self-administered instrument to assess levels of apathy in non-clinical individuals.

The Italian version of AMI may represent a suitable questionnaire to evaluate levels of apathy and motivation in healthy individuals for the following reasons: (a) unlike other scales of apathy, it was developed specifically for the general population, and it includes items designed to evaluate a range of activities targeted for healthy adults; (b) it provides cut-offs for detecting mild, moderate, and severe apathy (1.5, 1.66, and 2.06, respectively); and (c) it shows good psychometric properties.

Our findings confirmed fair internal consistency of the scale and an acceptable level of discrimination, similar to the original study [32]. In contrast to the original version, the Italian version of the AMI included 17 items, as one item (item 6 of the original scale) was excluded due to its weak correlation with the total score and its poor level of discrimination. Nonetheless, the Italian version of the AMI confirmed the structure of the original scale, revealing the presence of three factors: emotional sensitivity (the ability to feel positive and negative emotions), behavioral activation (engaging in goal-directed behavioral activity), and social motivation (being able to engage in social interactions) [32].

Moreover, divergent and convergent validity was demonstrated by our correlational results: the strongest association was found between AMI and DAS scores, as expected, whereas significant but weak associations were found between AMI and PHQ-9, BIS-15, and MMQ-A. In particular, our finding of a weak but significant association between apathetic and depressive symptoms may provide indirect evidence that these are two dissociated syndromes, although they share some overlapping manifestations [44, 45]. With regard to the negative association between AMI and MMQ-A scores, this finding suggests that higher levels of apathy may be linked to perceived memory dysfunctions not only in people with dementia [46] but also in people with preserved cognitive functioning. This issue should be explored in future studies by employing a comprehensive neuropsychological battery to assess other cognitive domains. Furthermore, the link between apathy and impulsiveness suggests the possible co-existence of these two syndromes affecting motivation, thereby rejecting their conceptualization as merely opposite ends of a single continuum [47], as already found in clinical populations (i.e., Parkinson’s disease or progressive supranuclear palsy) [48–50].

Our results also showed that AMI scores were not predicted by age, education, or sex of participants, in line with a previous investigation in healthy participants [51]. Moreover, the non-significant effect of formal education, the most common proxy of cognitive reserve, may suggest that not every proxy of cognitive reserve is equally associated with lower levels of apathy in healthy people, but this correlation may be found only for specific cognitive stimulating activities related to cognitive reserve, such as social and leisure activities [18]. Future studies could further explore the role of other sociodemographic and/or psychological variables (i.e., personality characteristics and/or several proxies of cognitive reserve) in apathetic behaviors. Finally, our cut-offs for detecting mild, moderate, and severe apathy in our Italian sample do not seem to differ significantly from the original English scale; this may be due to the relatively low cultural proximity between Italy and the UK. Future studies should focus on the possible differences in emotional sensitivity, behavioral activation, and social motivation in healthy adults living in countries with various degrees of cultural proximity.

One limitation of the present study is related to the sampling strategy, and a sampling bias could not be excluded; even if participants were from northern, central, and southern regions of Italy, most of the sample was from Southern Italy, and since the survey was disseminated via an online link, people with internet access problems may have been underrepresented. Finally, it should be noted that the validation of the present scale was not performed in a clinical setting, but the participants were asked to complete the behavioral scales by themselves at home. Therefore, future studies should evaluate possible differences in AMI scores across different settings (clinical vs. non-clinical settings).

In conclusion, the proposed cut-offs of AMI for mild, moderate, and severe apathy may help researchers and clinicians to identify people with lower levels of motivation and higher levels of apathy in the general population, which should be addressed by specific and tailored psychosocial and/or psychotherapy interventions to increase their emotional sensitivity, behavioral activation, and social motivation, and to reduce the risk of cognitive decline in later stages of life.

## Supplementary Information

Below is the link to the electronic supplementary material.

Supplementary file1 (PDF 199 KB)

Supplementary file2 (XLSX 11 KB)

## Data Availability

The data that support the findings of this study are available from the corresponding author upon reasonable request.
