# Supplementary material for: Evaluation of apathy in non-clinical populations: validation, psychometric properties, and normative data of the Italian version of Apathy-Motivation Index (AMI)
Source: Neurol Sci. 2023 Apr 3;44(9):3099–106. doi: 10.1007/s10072-023-06774-0 (PMC10415455; doi:10.1007/s10072-023-06774-0)
Supplement: Supplementary file 1 — Supplementary file1 (PDF 199 KB) [file 10072_2023_6774_MOESM1_ESM.pdf]

APATHY MOTIVATION INDEX (AMI) – versione italiana

Qui di seguito ci sono una serie di affermazioni. In ogni affermazione ti viene chiesto di pensare alla tua vita **nelle ultime 2 settimane**. Seleziona quanto ogni affermazione descrive la tua vita in questo periodo. Barra "Completamente vero" se l'affermazione ti descrive perfettamente, "Completamente falso" se l'affermazione non ti descrive per niente nelle ultime 2 settimane, e usa le risposte intermedie di conseguenza.

	Completamente FALSO	Abbastanza falso	Né vero né falso	Abbastanza vero	Completamente VERO
1. Mi sento triste o turbato quando ricevo delle brutte notizie.	☐	☐	☐	☐	☐
2. Do inizio alle conversazioni con le persone che non conosco.	☐	☐	☐	☐	☐
3. Mi piace fare qualcosa con persone appena conosciute.	☐	☐	☐	☐	☐
4. Propongo ai miei amici attività che possiamo svolgere (insieme).	☐	☐	☐	☐	☐
5. Prendo le decisioni con fermezza e senza esitazioni.	☐	☐	☐	☐	☐
6. Basandomi sulle ultime due settimane, direi che mi sta molto a cuore ciò che le persone che mi vogliono bene pensano di me.	☐	☐	☐	☐	☐
7. Esco con i miei amici ogni settimana.	☐	☐	☐	☐	☐
8. Quando decido di fare qualcosa, riesco ad impegnarmi facilmente.	☐	☐	☐	☐	☐
9. Non mi piace stare senza far niente.	☐	☐	☐	☐	☐
10. Faccio le cose quando vanno fatte, senza che gli altri debbano ricordamelo.	☐	☐	☐	☐	☐
11. Quando decido di fare qualcosa, sono motivato a farla fino alla fine.	☐	☐	☐	☐	☐
12. Mi sento male se dico qualcosa di insensibile/indelicato.	☐	☐	☐	☐	☐
13. Inizio una conversazione senza essere spinto a farlo.	☐	☐	☐	☐	☐
14. Quando devo fare qualcosa, la faccio subito, così mi tolgo il pensiero.	☐	☐	☐	☐	☐
15. Mi dispiace quando sento che un conoscente ha avuto un incidente o si è ammalato.	☐	☐	☐	☐	☐
16. Mi piace scegliere cosa fare tra varie attività.	☐	☐	☐	☐	☐
17. Se mi rendo conto di non essermi comportato bene con qualcuno, dopo mi sento terribilmente in colpa.	☐	☐	☐	☐	☐

Developed by Ang. et al (2017). Italian translation by Altieri et al. (2023)

APATHY MOTIVATION INDEX (AMI) – versione italiana

Istruzioni per l'attribuzione dei punteggi.

Per l'attribuzione dei punteggi è necessario assegnare il punteggio degli item secondo questo schema:
Completamente VERO = 0
Abbastanza vero = 1
Né vero né falso = 2
Abbastanza falso = 3
Completamente FALSO = 4

Ogni punteggio delle singole sottoscale è dato dalla media degli item del dominio specifico. Il punteggio medio va da 0 a 4 (0 corrisponde ad essere motivati e 4 corrisponde ad essere apatici).

(1) Scala di attivazione comportamentale (apatia comportamentale): Item 5,8,9,10,11,14
(2) Motivazione sociale (apatia sociale): Item 2, 3, 4, 7, 13, 16
(3) Sensibilità emotiva (apatia emotiva) : Item 1, 6, 12, 15, 17

Il punteggio totale dell'AMI è dato dalla media delle 3 sottoscale.
